# Polymer Nanocomposite Containing Palladium Nanoparticles: Synthesis, Characterization, and Properties

**DOI:** 10.3390/polym14183795

**Published:** 2022-09-10

**Authors:** Gleb Yurkov, Yury Koksharov, Alexander Fionov, Nikolai Taratanov, Vladimir Kolesov, Vladislav Kirillov, Mstislav Makeev, Pavel Mikhalev, Dmitriy Ryzhenko, Vitaliy Solodilov

**Affiliations:** 1 N.N. Semenov Federal Research Center of Chemical Physics of Russian Academy of Sciences, 119334 Moscow, Russia; 2 Department of Structurally Sensitive Functional Materials, Bauman Moscow State Technical University, BMSTU, 2-nd Baumanskaya, 5, 105005 Moscow, Russia; 3 Faculty of Physics, M.V. Lomonosov Moscow State University, 119991 Moscow, Russia; 4 Kotelnikov Institute of Radio Engineering and Electronics of Russian Academy of Science, 125009 Moscow, Russia; 5 Ivanovo Institute of State Fire Service of Emercom of Russia, 153040 Ivanovo, Russia

**Keywords:** polymer composite, nanoparticles, palladium, EPR spectra, EXAFS, electrophysical properties

## Abstract

Composite nanomaterials have been prepared through thermal decomposition of palladium diacetate. The composite contains palladium nanoparticles embedded in high-pressure polyethylene. The materials were studied by a number of different physico-chemical methods, such as transmission electron microscopy, X-ray diffraction, X-ray absorption spectroscopy, electron paramagnetic resonance, and EXAFS. The average size of the nanoparticles is 7.0 ± 0.5 nm. It is shown that with the decrease of metal content in the polymer matrix the average size of nanoparticles decreased from 7 to 6 nm, and the coordination number of palladium also decreased from 7 to 5.7. The mean size of palladium particles increases with the growing concentration of palladium content in the matrix. It is shown that the electrophysical properties of the material obtained depend on the filler concentration. The chemical composition of palladium components includes metallic palladium, palladium (III) oxide, and palladium dioxide. All samples have narrow lines (3–5 Oe) with a g factor of around two in the electron paramagnetic resonance (EPR) spectra. It is shown that EPR lines have uneven boarding by saturation lines investigation. The relaxation component properties are different for spectral components. It leads to the spectrum line width depending on the magnetic field value. At first approximation, the EPR spectra can be described as a sum of two Lorentzian function graphs, corresponding to the following two paramagnetic centers: one is on the surface, and one is inside the palladium particles. Some of the experimental characteristics were measured for the first time. The data obtained indicate interesting properties of palladium-based nanocomposites, which will be useful for obtaining products based on these materials.

## 1. Introduction

Nanotechnology is an expanding part of peoples heritage. Its main applications are the development of innovative methods to fabricate new products, the formulation of new chemicals and materials, and improving the performance of the current generation of equipment with newer ones. As a result of this activity, the following features are targeted: lower consumption of materials and energy and decreased harm to the environment, as well as some environmental remediation [1]. Examples of the above methods’ applications can be quantum [2], and molecular assemblies based on nanotechnology, i.e., systems that are able to achieve assembly-line-like production of molecules [3].

The ways to produce nanomaterials with expected properties are currently under investigation in many scientific centers around the world [4,5,6,7,8]. The majority of the materials are composite materials with nano-sized and nanostructured components (nanotubes, nanofibers, etc.) [9]. The introduction of nanoparticles into a polymer matrix could increase relative permittivity or breakdown strength, which is crucial for energy storage devices [10]. A method that consists of dispersing insoluble fillers such as nanoparticles of high strength and stiffness into an epoxy resin is used to increase the critical stress intensity factor and the critical strain energy release rate to improve epoxy toughening [11]. It is known that metal nanoparticles have catalytic activity when used in chemical and photographic processes [12,13].

Palladium absorbs large volumetric quantities of hydrogen at room temperature and ambient pressure, making the palladium hydride system a promising candidate for hydrogen storage [14]. Materials with palladium may be used not only for storage devices but for other applications, such as a catalyst for hydrogen production [15,16]. Water electrolysis is considered to be a particularly promising alternative since it only uses water as a reactant. Researchers have attempted to enhance the performance of water electrolysis by optimizing the electrolytes, catalysts, and working conditions. The development of high-performance catalysts for the hydrogen evolution reaction in water electrolysis is very important to be able to achieve efficient hydrogen production [17]. Hydrogen fuel cells, being hydrogen energy storage devices, are the most effective and environmentally friendly means of energy accumulation and storage. Direct current provides the accumulation of electric energy and is therefore necessary when using renewable energy sources [18]. Nanosized metal catalysts have improved properties compared to commercial ones. For example, a catalyst structure is identified and the size of distributed metal nanoparticles is in the narrow range of 2–3 nm; the mass activity of the prepared catalyst is over 1.5 times higher in fuel cell testing than that of a commercial catalyst [19]. Palladium is used as a catalyst in organic reactions such as dye reduction [20], alcohol oxidation [21], and catalytic oxidation [22].

Palladium is an interesting metal from a fundamental point of view too. As a bulk metal, it has paramagnetic behavior at 300 K while its magnetic susceptibility equals 8 × 10^−4^ [23]. A higher volume of the susceptibility of palladium compared to other noble metals can be explained by the higher electronic density of states at the Fermi edge [24]. The electronic structure of palladium nanoparticles is susceptible to the inner (size, morphology, structure) and outer (ligands, charge) factors [23]. Electronic paramagnetic resonance (EPR) spectroscopy is widely used for nanoparticle structure investigation [25]. EPR spectra of palladium nanoparticles have been investigated in a wide range of sizes (typical size *d* ≈ 2.5 nm) [26], *d* ≈ 20–40 nm [27], *d* ≈ 40 nm [28], *d* ≈ 35–110 nm [29]. The EPR spectra have not been found for Pd nanoparticles with an average diameter of 7–10 nm. The particle of such size is large enough for the “magic numbers” effect to be negligible [26]. On the other side, it is small enough to have quantum-surface effects [30].

Systems based on palladium nanoparticles are of growing interest and can be used for various purposes, such as antimicrobial protection, catalysis, and wastewater treatment [31,32,33,34]. It is also noteworthy that palladium nanoparticles can be successfully synthesized using green synthesis technology.

Electrodes made of materials modified with palladium nanoparticles can be used for photoelectrochemical removal of organic pollutants in wastewater [33]. Moreover, palladium nanoparticles are able to bind to the walls of bacterial cells and penetrate through their membranes, causing the destruction of bacterial cells and leakage of intracellular components [31].

In addition, palladium is a widely used catalyst in industry, in particular, for the hydrogenation of unsaturated hydrocarbons. Therefore, it is of great interest to manufacture industrial catalysts based on palladium nanoparticles, in which both surface and bulk structures exhibit a dynamic nature under reaction conditions [32,34].

The aim of this work is the development of a method for the synthesis and study of a composite nanomaterial that consists of palladium-containing nanoparticles stabilized in the high-pressure polyethylene (HPPE) matrix. Some experimental results, in particular from the EPR study, were presented for the first time. Due to this, a more complete understanding of the properties of the resulting material was obtained, which can be useful for the manufacture of devices based on it.

## 2. Materials and Methods

### 2.1. Materials

Acetic (glacial acetic) (CH_3_COOH, 99.5%) and nitric acids (HNO_3_, 90+%), sodium borohydride (NaBH_4_, 98+%), chloroform (trichloromethane, CHCl_3_, 99+%), hexane (C_6_H_14_, 95+%) were purchased from Acros Organics (Geel, Belgium) and used without further purification. Commercially available high-pressure polyethylene (HPPE) and mineral oil were used.

### 2.2. Preparation of Precursor and Composites

Palladium diacetate (palladium (II) acetate, *Pd(CH_3_COO)_2_*) was added to the solution—the melt of high-pressure polyethylene. Chloroform was used as solvent. Prior to the synthesis Palladium diacetate was synthesized from *Pd* black by oxidation with glacial acetic acid in presence of nitric acid as follows:

*Pd* + 2*HNO*_3_ + 2*CH*_3_*COOH* → *Pd*(*CH*_3_*COO*)_2_ + 2*NO
*_2_ +2*H*_2_*O* (1)

Preparation method of palladium diacetate is described here. The first step is the affinage of *Pd* black. One-tenth part (1/10) of the initial sample of *Pd*-black (3 g) is placed in a thermostable 250 mL volume glass reactor (Mettler Toledo, Greifensee, Switzerland) with 30 mL of aqua regia (*HCl* cond.: *HNO* 3con. = 3:1 vol.) and is heated until a bright red coloration is observed. Then the reactor is removed from the hotplate. The rest of the palladium black is added to the reaction mixture in small portions. The water is added to the mixture until the volume equals 150–200 mL after the dissolution of the palladium black. The pH of the reaction mixture is increased up to 12 by adding small portions of a potassium hydroxide solution. The brown precipitate of *Pd*(*OH*)_2_ is formed. The palladium hydroxide is reduced by sodium borohydride to metal palladium. The sodium borohydride is added in small portions until the discoloration occurs and the palladium is formed.

The palladium black is washed several times with distilled water till the total absence of chloride ion is achieved (usually it takes a portion of 600–700 mL of water and 7–8 iterations). The sample is washed with 100–150 mL of glacial acetic acid 3 times.

The purified palladium black is moved to a thermostable round bottom flask (of around 100 mL volume). Then 1 mL of concentrated *HNO*_3_ is added to the flask. The reaction mixture is heated and boiled for 2–3 h till brown gas (*NO*_2_) release terminates. The scheme of thermal decomposition is presented in our earlier work [35].

The obtained red-brown solution is filtered through the DURAN^®^ filter crucible (Duran, Mainz, Germany), porosity 4, and evaporated at a rotary evaporator till the residue solution volume is 1/3 or 1/4 of the initial volume. The solution is cooled and a precipitate is formed. The precipitate is filtered through the filter crucible porosity 2 or 3 and dried in a glass vacuum desiccator by alkali. The formation of the palladium diacetate is proven by IR spectroscopy.

The nanoparticle synthesis in a polyethylene matrix was carried out by the methods already described for a variety of metals [36,37,38]. The thermal decomposition of palladium diacetate dissolved in chloroform was performed at a constant temperature of 300 °C under argon atmosphere and vigorous mixing. Metal-containing component (MCC) was dropwise introduced into the reaction mixture (polyethylene (PE)—oil solution melt) by a dropping funnel at the rate of 30 mL/h. Gaseous products were removed by the flow of argon. The inert gas rate was adjusted to ensure fast removal of the gaseous products. The reaction mass was held for 1 h at the synthesis temperature under vigorous mixing after the addition of the entire volume of MCC. The reaction mixture was cooled to room temperature under argon atmosphere. The samples were deoiled with hexane in a Soxhlet extractor, then dried, and stored at ambient temperature and air. Palladium contents in the sample were 5, 10, and 20 wt.%. The samples had the appearance of black powder.

### 2.3. Equipment and Measurement

X-ray powder diffraction was recorded at Shimadzu XRD 6000 (CuK_α_ radiation, λ = 154,056 Å, graphite monochromator. The obtained diffraction patterns were compared to the PDF2 of the Joint Committee on Powder Diffraction Standards.

The relation between the particle size and the width of the diffraction line at half maximum intensity was proposed by Scherrer [39] and the assumption that the radiation was parallel and the sample was transparently allowed us to employ the following equation:

(2) $$d=\frac{K\lambda }{\beta cos\theta }$$

where *d* is the average size of particles, *λ* is the X-ray wavelength, *β* is the line broadening at half the maximum intensity (FWHM), *K* is a dimensionless shape factor, with a value close to unity. It is proportional to the ratio of FWHW for Gaussian function and integral breath IB (the area of the Gaussian function beneath the maximal value of the function). K can be calculated by $K=2\sqrt{\ln\left(\frac{2}{n}\right)}\equiv \frac{{\mathrm{FWHM}}_{G}}{{\mathrm{IB}}_{G}},$ *θ* is a diffraction angle. It is known that the methods of particle size determination have limitations due to different and unknown shapes of particles [40,41].

Transmission electron microscopy (TEM) was applied to conduct additional particle size determination. JEOL JEM-1011 microscope was used with 80 kV acceleration voltage. The sample was dispersed in hexane under ultrasound radiation for the TEM investigation. A copper wafer was coated with the prepared mixture, a special resin coat, and carbon.

The room temperature EPR spectra have been recorded using the X-band Varian E-4 computerized spectrometer. The method “peak-to-peak” was used to measure the width and amplitude of the resonance lines. The amplitude modulation of 1 Oe was used. The saturation effects have been studied mostly at microwave power from 0.5 up to 200 mW. The typical sample mass has been in the range of 5–10 mg.

EXAFS spectrometer at the structural material science station of the Kurchatov Synchrotron Centre (Moscow) with electron-beam energy of 2.5 GeV and a current of 80 mA was used to research the electronic and atomic structure of Pd nanoparticles by X-ray absorption spectroscopy. X-ray Pd K absorption edges were measured in the transmittance stance. X-ray radiation was corrected by a double crystal Si(111) monochromator. Processing of the spectra included background discrimination, normalization to the K edge jump, and isolation of atomic absorption μ_0_. EXAFS χ spectra were analyzed by Fourier transformation in the interval of photoelectron wave vectors k = 3.0–14.0 Å^–1^ with weight function k^3^. Radial distribution of atoms of the nearest coordination spheres (CS) with some phase correction could be attributed to Fourier transform moduli (FMTs). The maximum of the first derivative of the K edge was chosen for a threshold ionization energy E_0_ value determination. The threshold ionization energy varied in subsequent corrections.

Nonlinear fitting of the structural parameters (interatomic distances, coordination numbers (CNs), Debye-Waller factor (*σ^2^*) of the corresponding CSs in comparison to the calculated EXAFS signal and the signal isolated from the full EXAFS by Fourier filtering were used for the quantitative characteristics of the nearest neighboring environment surrounding palladium atoms in the samples. The program packet IFFEFIT-1.2.11 was used for the calculations. FEFF7 was used for calculation of the phases and scattering amplitudes for plotting the model spectrum. The bulk palladium data of atomic coordinates were used.

The bulk samples were prepared by the hot pressure method of the heated sample for electro-physical investigation. A steel mold was heated in a muffle furnace up to temperature *T_heat_* = 250 °C with heating rate of 10°/min. The sample was sintered for 30 min at constant temperature. The mold was transferred into a manual press with the pressure of 6 kH. The powder samples were changed from powder to visco-fluid state. The cooling of the samples was performed in the press by air, without additional chilling, under a pressure of 6 kH. The pressed samples were prepared as tablets measuring 26 mm in diameter and 1.5 mm in thickness. The tablets were homogenous with smooth surfaces. The E4980A precision LCR meter (Keysight Technologies) was used. The dielectric constant and the dielectric loss tangent tg*δ* (at frequency 1–1000 kHz) and the specific volume resistance *ρ_V_* of the samples were measured by a two-electrode measuring cell—a capacitor. The electrical resistivity of the cell with the sample was measured by the 4339B high-resistance meter (Keysight Technologies, Santa Rosa, CA, USA). The samples were investigated in a shielded metering camera in the resistance range of 10^3^––10^18^ Ohm (Ω) with a measuring voltage of 10—1000 V.

Bruker system for magnetic field control and Sartorius electronic scale were used for magnetic susceptibility. The maximal value of the magnetic flux density was 6.5 kG.

The quasi-optical technique was used for reflection and shielding measurements of the nanocomposite samples. Equipment for measuring the standing wave ratio (SWR or voltage standing-wave ratio) was used at the reflection frequency of 30 GHz. The method was based on measuring the reflection coefficient and attenuation coefficient of the electromagnetic wave in a tract equipped with directed branches. P2–65 by NPP-Elmika equipment was used with a tract (width × high = 7.2 × 3.4 mm), and the frequency range 25.95–37.5 GHz. The measurement cell was made of two correlated emitting horns connected to the waveguide of the meter. The sample was placed between emitting and receiving horns. The attenuation coefficient and reflection coefficient for the tested material were respectively calculated based on the measurement at the fixed wave of 30 GHz. The Standing-wave ratio was no more than 1.1.

## 3. Results and Discussion

**Figure 1:**
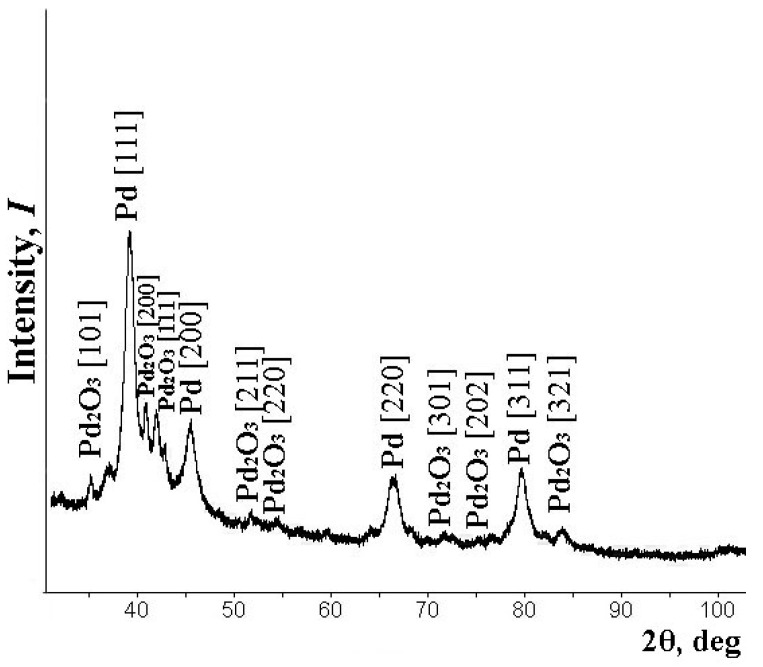
Typical X-ray diffraction samples of the nanocomposite.

A series of composite powder materials with palladium were synthesized. The metal content was 5, 10, and 20 wt.%. All the samples were investigated according to the described above procedures. X-ray powder diffraction analyses were used for the determination of the phase composition of the prepared palladium diacetate samples. The characteristic reflections of *Pd* and *Pd_2_O_3_* are presented in the spectra. All reflection maxima are in good agreement with the PDF2 data [42]. It attests that all particles have a well-formed crystalline structure. A typical X-ray diffraction pattern for synthesized and investigated samples is shown in Figure 1.

**Figure 2:**
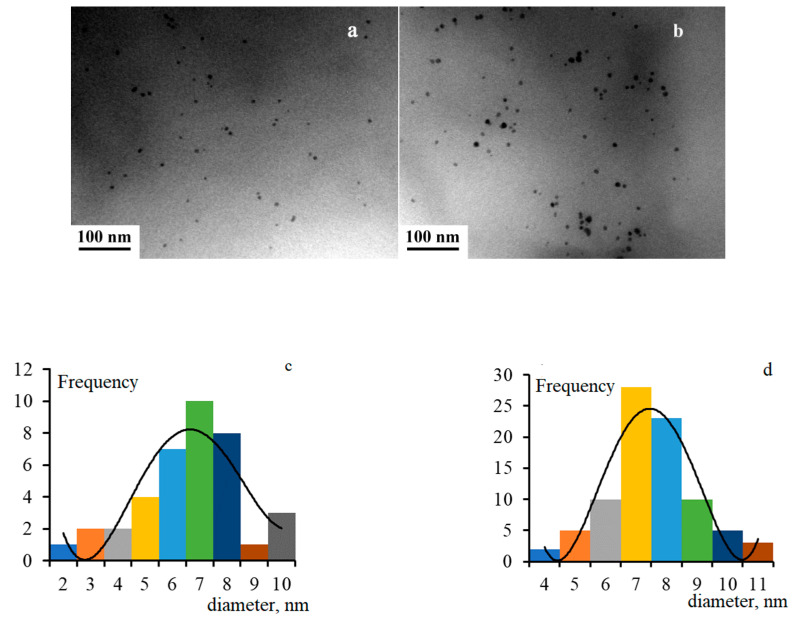
Microscopic image of the nanocomposite with 10 wt.% *Pd* (**a**) and 20 wt.% *Pd* (**b**) and their size distribution (**c**,**d**, respectively).

The average size of *Pd* nanoparticles has increased from 6.5 nm to 7.5 nm with an increase in the metal concentration according to the TEM results. The same range of sizes is calculated by (2). The typical microscopic images of the nanocomposite and their size distribution are shown in Figure 2.

Some structural characteristics of the samples with between 10 and 20 wt.% metal content are described in the previous short article [36]. The EXAFS method makes it possible to obtain averaged information on the local environment of all-absorbing atoms of the element and, unlike XRD, the EXAFS method is suitable for the analysis of nanoscale systems [43]. The data obtained are shown in Table 1.

The amplitude of the main peak at r = 2.50 Å (without phase shift) is the highest in the metallic foil and the lowest in the nanocomposite with 10 wt.% *Pd*. It corresponds to the shortest *Pd*–*Pd* distance in the face-centered cubic (FCC) structure. It was proposed that *Pd* is oxidized during synthesis or storage. *Pd* small particles tend to oxidize in atmospheric air, as shown in the example for catalyst activity [43]. A model of nanoparticles formed from a mixture of metallic palladium and palladium oxide has been constructed. The CS radius in nanoparticles, which corresponds to the *Pd*–*Pd* distance, agrees (within the limit of the experimental error bracket) with the value type of the fcc structure. In the palladium foil, the coordination number is higher than for *Pd* samples of between 20 and 10 wt.% (12, 7, and 5.7 respectively). It is correlated with small palladium particles found in the above-mentioned catalyst activity [43]. The samples of *Pd* 10 wt.% and *Pd* 20 wt.% have the radii of the nearest CSs consisting of oxygen atoms between 1.93 and 1.96 Å. It is very close to between 1.94 and 1.95 for small *Pd* particles [43] or to the radius in mixed palladium oxide of *Pd*^2+^ and *Pd*^4+–^*KPd*_2_*O*_3_ (1.963(9) Å [44]. The stable *PdO* has a bigger radius (~2.10 Å).

**Figure 3:**
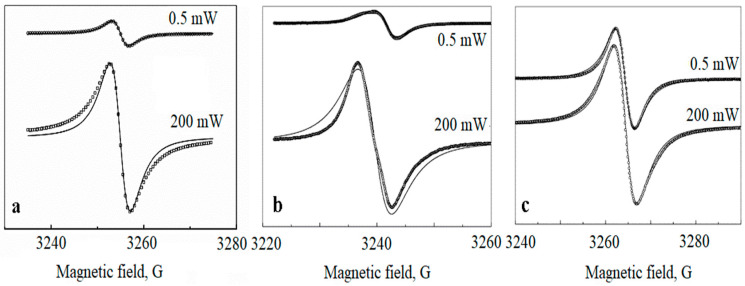
Room temperature EPR spectra of *Pd* nanoparticles in polyethylene matrix for different metal content: 5 wt.% (**a**), 10 wt.% (**b**), 20 wt.% (**c**). The spectra for low and high microwave power values (0.5 mW and 200 mW) are shown. The solid lines are fitting Lorentzians, the circle (light) lines are the experimental curves.

The EPR spectra have been collected for samples with a different metal content as follows: 5, 10, and 20 wt.%. The typical room temperature EPR spectra are shown in Figure 3 for low (0.5 mW) and high (200 mW) values of the microwave power.

Experimental data for the pure polymer matrix are presented in Appendix A1.

**Figure 4:**
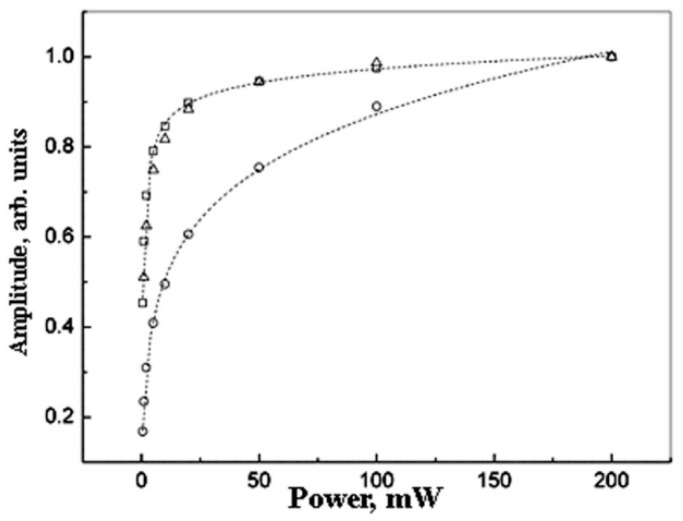
The saturation effect for the normalized EPR amplitude for *Pd* nanoparticles for different metal content: 5 wt.% (circles ○),10 wt.% (squares □), and 20 wt.% (triangles ∆). The dot lines show the fitting by formulae (3).

**Figure 5:**
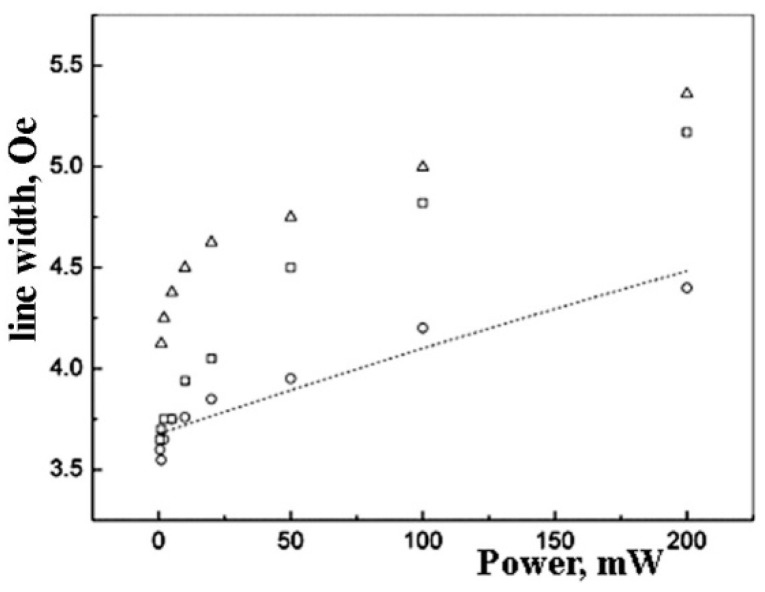
Linewidth saturation curves for experimental EPR spectra for the *Pd* nanoparticles in polyethylene for the concentrations of 5 wt.% (○), 10 wt.% (□), and 20 wt.% (∆). The dotted line showed the approximation by formulae (4) for 5 wt.%.

It is clear the experimental spectrum shape deviates from the Lorentzian. This deviation can be due to the inhomogeneous broadening of the spectra. To check this possibility, the saturation effects have been investigated (Figure 4 and Figure 5). It is known that homogeneous and inhomogeneous EPR lines differ in their saturation behavior [45,46].

The amplitude saturation law is near equal for samples with *Pd* content 10 wt.% and 20 wt.% (Figure 4).

This law can be written [45] as follows:

(3) $$A=C\sqrt{P{\left(1+P/P_{0.5}\right)}^{b/2}}$$

where *C* is the power-independent parameter, *P* is the microwave power value, *P*_0.5_ is the characteristic power value, and *b* is the inhomogeneity parameter [46]. The value of *b* is equal to 3 for the ideal homogeneous line and 1 for the ideal inhomogeneous line.

The parameter *b* value is maximal for the samples of 20 wt % and minimal for the samples of 5 wt.% *Pd*. This correlates with the minimal deviation of the EPR spectrum shape from the Lorentzian for the samples of 20 wt % (Figure 3).

The EPR linewidth is larger for the samples with higher metal content (Figure 5). This effect is more distinct at higher microwave power values.

For the sample of 5 wt% *Pd*, the saturation law for the linewidth can be fitted by the formulae [46].

(4) $$∆H=\sqrt{∆H_{0}^{2}+4T_{1}kQP/T_{2}}$$

where Δ*H* is the linewidth, **Δ***H_0_* is the linewidth at the lowest microwave power, *k* is the numerical coefficient (4 × 10^−4^ in our calculation), *Q* is the resonator quality-factor of about 3500, *T_1_* and *T_2_* are relaxation times. We have found that the ratio of relaxation times is around 200, and **Δ***H_0_* is close to 4. (**Δ***H_0_* = 3.7 ± 0.1; *T_1_*/*T_2_* = 180 ± 20).

**Figure 6:**
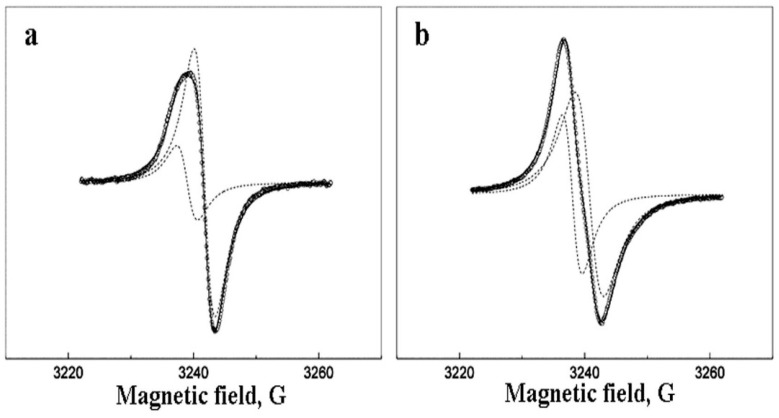
The numerical decomposition of the EPR spectra for the sample with 10 wt.% *Pd* at 1 mW (**a**) and 100 mW (**b**). The circles are experimental points, the dotted lines are Lorentzian components, solid line is the sum of these Lorenzians.

**Figure 7:**
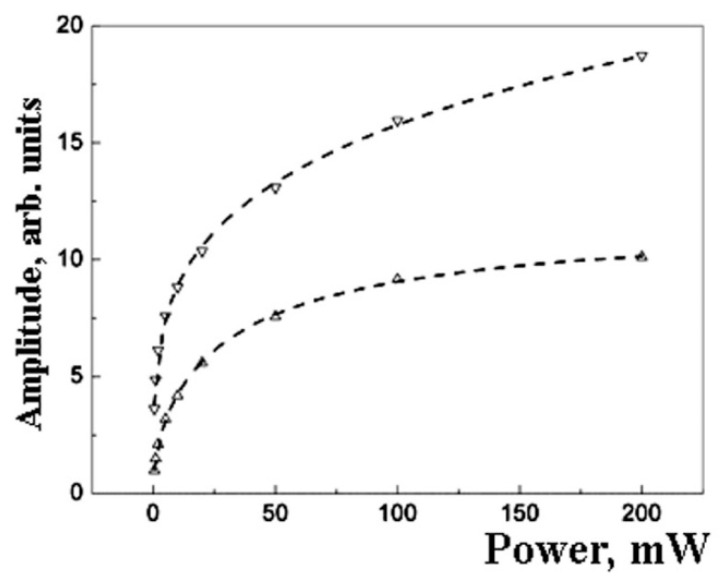
The amplitude saturation curves for the two Lorentzians presented at Figure 6. The dot lines show approximation by formulae (3). The upper line has the following parameters: *P*_0.5_ = 0.3 ± 0.2; *b* = 0.50 ± 0.02. The bottom line has *P*_0.5_ = 60 ± 15; *b* = 1.0 ± 0.1.

**Figure 8:**
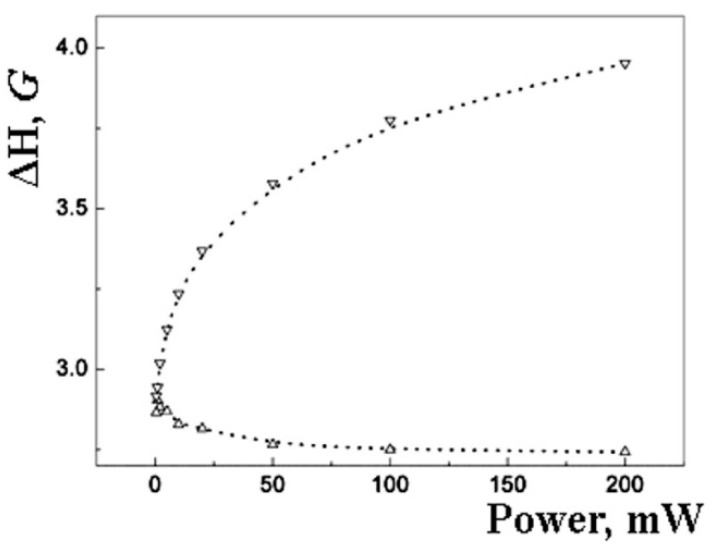
Dependence of the width of the EPR signal components (Lorentians) of palladium nanoparticles in a sample with 10 wt% microwave power (room temperature). Circles are experimental points; dotted lines are parameters of the calculated Lorentzians.

The deviation of the EPR line from the Lorentzian may be due to the presence of two (or more) types of EPR active centers in nanoparticles. Figure 6 shows the decomposition of EPR spectra into two Lorentzians for the sample with 10 wt.% *Pd*. Figure 7 and Figure 8 show the related saturation curves for this sample. The parameters of these curves indicate an inhomogeneous type of saturation for both components.

It is possible that one of the Lorentzians in the spectra decomposition corresponds to the core of the *Pd* nanoparticle. The second

Lorentzian can be related to the surface of the particle. The core and surface centers should have different relaxation properties. It can result in a difference in EPR saturation behavior (Figure 7 and Figure 8).

The tablets have been prepared by the hot-pressed method. The sizes and masses of the tablets are shown in Table 2.

The values are calculated by the volumes and masses of the tablets for the densities of the samples. The specific gravity of the palladium-containing composites increases with the metal concentration increase. The density of unfilled polyethylene after the reaction process is 0.914 g/cm^3^ as measured by the hydrostatic method.

The results of the dielectric permeability (ε) measurement and the loss tangent (tgδ) (the loss reaction to the electric field) are shown in Table 3 at frequencies 1–1000 kHz. The specific volumetric electrical resistances are shown as well.

**Figure 9:**
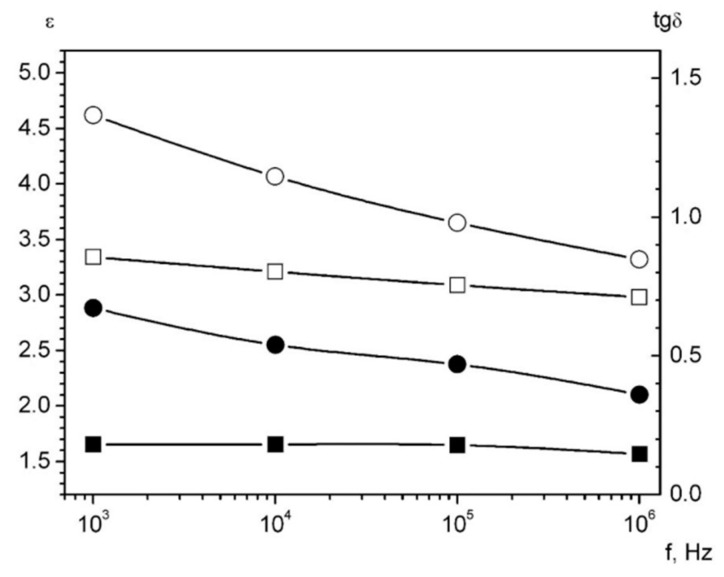
The dielectric permeability and the loss tangent for the composites. Black symbols represent tg*δ*. The white symbols show *ε*. The squares are for the sample *Pd* 10 wt%, the circles are for the sample *Pd* 20 wt%.

The dependence on frequency for dielectric permeability and loss tangent is shown in Figure 9 for the palladium-containing composites.

The strongly pronounced frequency dependence of ε and tg*δ* of palladium-containing composites testifies to the presence of the slow polarization mechanisms, which could be caused by inhomogeneous core-shell nanoparticles.

The specific volumetric resistivity when the concentration of nanoparticles from 10 to 20 wt% decreases by three

Orders of magnitude but remains high, characteristic of polymer dielectrics. This decrease in ρV may be caused by the tunneling and jumping mechanisms of electrical conductivity occurring in composites based on metal-containing nanoparticles [47,48,49,50]. It should be mentioned that the distinct dependence of dielectric permittivity and relative volume resistivity on metal concentrations occurred for all palladium content in the nanocomposites.

### Properties of the Samples in the Microwave Range

The reflection and attenuation coefficients of the composite samples have been measured by the quasi-optical technique using an R2–65 m that measures the voltage standing wave ratio and attenuation in the waveguide at frequencies of 30 GHz. The power of electromagnetic waves that are reflected, transmitted, or absorbed by a dielectric layer can be estimated using the law of energy conservation as follows:

*P_inc_* = *P_it_* + *P_refl_* + *P_loss_* (5)

where *P_inc_*, *P_tr_,* and *P_refl_* are incident, transmitted, and reflected powers, and *P_loss_* is the lost power in the dielectric material. Attenuation coefficient *A* is measured as the ratio of the microwave power that has passed through the cell with the sample to the incident power as follows:

(6) $$A=-10\log\frac{P_{tr}}{P_{inc}}\;\left[dB\right]$$

Reflection coefficient *R* in relative units is calculated by the following formulae:

(7) $$R=\frac{P_{refl}}{P_{inc}}.$$

Relative loss factor *L* is calculated as follows:

(8) $$L=\frac{P_{loss}}{P_{inc}}.$$

The results of the measurements are shown in Table 4 as characteristics for attenuation (*A*), reflection (*R*), loss (*L*).

A decrease in the transmittance coefficient with an increase in reflection coefficient has occurred with increasing metal concentration. The loss coefficient is within the range of 3–10%.

*M_s_*(*B*) = *A* + *C* × *B* (9)

**Figure 10:**
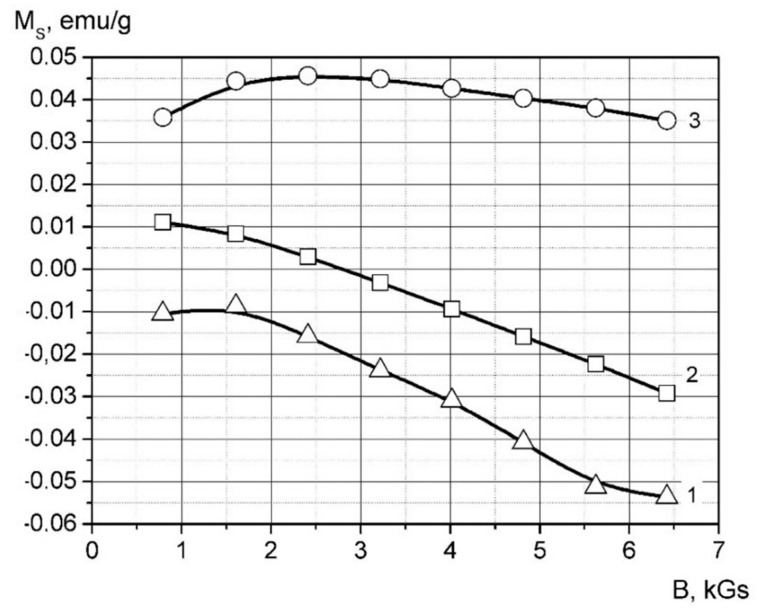
Magnetization curves the palladium containing composites. 1-HDPE, 2-the sample HDPE + 10 wt.% *Pd*, 3-the sample HDPE + 20 wt.% *Pd*.

The magnetization curves of the samples are shown in Figure 10. A specific magnetic susceptibility has been calculated by parameter *C* from the linear regression of the magnetization curve in the field of 2.5–6.5 Gs as follows:

Obtained data for magnetic susceptibility are shown in Table 5.

It is shown from the data that introducing palladium nanoparticles into the matrix leads to a prominent magnetic susceptibility decrease. The difference between matrix susceptibility and composite is increased with higher filler content. This result is typical for paramagnetic material, which is palladium *χ_rel_* = 5.33 × 10^−9^ m^3^/kg [51].

## 4. Conclusions

The possibility of using the thermal decomposition of palladium-containing compounds to obtain palladium-containing nanoparticles in a polyethylene matrix was demonstrated. The resulting materials, using palladium diacetate as a precursor, were shown to have palladium nanoparticles with an average diameter of 6–10 nm. It has been found that nanoparticles produced by metal-containing compound decomposition at 300 °C have metallic palladium and oxide palladium phases. The chemical composition (*Pd* and *Pd*_2_*O*_3_), nanoparticle size, and particle interaction with the matrix were determined by a complex of methods. Some of the experimental characteristics were measured for the first time. The data obtained indicate interesting properties of palladium-based nanocomposites, which will be useful for obtaining products based on these materials. It is shown that with the decrease of metal content in the polymer matrix the average size of nanoparticles decreased from 7 to 6 nm, and the coordination number of palladium also decreased from 7 to 5.7.

The results of an EPR study of palladium nanoparticles in a polyethylene matrix indicate a highly inhomogeneous electronic structure of the particles. The possibility of decomposition of the EPR spectra into two components points to the possibility of separation of two main EPR-active phases in the particle structure. Since the differences in the content of the oxide and metallic components of nanoparticles at different particle concentrations in the matrix are detected, it can be assumed that the two-component composition of the EPR is responsible for the bulk and surface phases of the nanoparticles. The detected difference in the relaxation properties of the individual components of the EPR spectra indicates the possible manifestation of quantum-dimensional effects in them.

Insertion of palladium nanoparticles into a high-pressure polyethylene matrix leads to an increase in *ε* and tg*δ* as well as to a decrease in *ρ_V_* and *χ_rel_*. This concentration dependence is established for this relation. The strongly pronounced frequency dependence of ε and tgδ of palladium-containing composites testifies to the presence of slow polarization mechanisms, which may be caused by the core shell.

As the concentration increases, there is a slight decrease in the transmission coefficient and an increase in the reflection coefficient at a frequency of 30 GHz. At the same time, the loss factor is within 3–10%.

## Figures and Tables

**Table 1:** Structural data for the local atomic environment of *Pd* atoms obtained by two-sphere EXAFS fitting (*R* are the interatomic distances, *N* is the coordination number, *σ_2_* is the Debye-Waller factor, and *Q* is the fit quality function).

Sample	Phase	*N*	*R*, Å	*σ_2_*, Å	The Type	*Q*
*Pd*-foil	*Pd*_met_	12	2.75	0.0065	*Pd*-*Pd*	2.0 ^1^
PE-*Pd* (10 wt%)	*PdO* *Pd*_met_	1.8 5.7	1.93 2.75	0.0040 0.0067	*Pd*-*O* *Pd*-*Pd*	12 ^2^
PE-*Pd* (20 wt%)	*PdO* *Pd*_met_	1.7 7	1.96 2.74	0.0040 0.0067	*Pd*-*O* *PdO*-*Pd*	7.5 ^2^

^1^ Window r = 2.0–2.9 Å; ^2^ Window r = 1.0–3.0 Å.

**Table 2:** The sizes and masses of the tablets were produced by the hot-pressed method.

Sample	Composition	*d*, mm	*h*, mm	mass, g	*ρ*, g/cm^3^
HPPE + 10% *Pd*	PE + 10% *Pd*	25.8	1.82	0.960	1.008
HPPE + 20% *Pd*	PE + 20% *Pd*	25.8	1.47	0.850	1.104

**Table 3:** The dielectric permeability and the loss tangent for the composites and original PE.

Sample	*ρ_V_*, Om × m	*ε* and tg*δ* (in Brackets) at *f_work_*:			
		1 kHz	10 kHz	100 kHz	1 MHz
HPPE + 10% *Pd*	2.0 × 10^15^	3.34 (0.180)	3.21 (0.180)	3.09 (0.178)	2.98 (0.146)
HPPE + 20% *Pd*	3.2 × 10^12^	4.62 (0.672)	4.07 (0.539)	3.65 (0.469)	3.32 (0.360)
PE	1.7 × 10^16^	2.36			2.25

**Table 4:** Attenuation, reflection, and loss coefficients of the palladium nanoparticles samples.

Sample	*F_work_* = 30 GHz		
	*A*, dB	*R*	*L*
HDPE	0.3	0.12	0.0
HDPE + 10% *Pd*	1.05	0.12	0.09
HDPE + 20% *Pd*	1.10	0.19	0.03

**Table 5:** Parameters of linear regression and magnetic susceptibility.

Sample	*C*, cm^3^/g	*χ_rel_* (Experiment), 10^−9^ m^3^/kg
HDPE	(−9.5 ± 1.1) × 10^−3^	−0.75 ± 0.09
HDPE + 10% *Pd*	(−7.9 ± 0.2) × 10^−3^	−0.63 ± 0.02
HDPE + 20% *Pd*	(−2.8 ± 0.4) × 10^−3^	−0.23 ± 0.03

**Figure A1:**
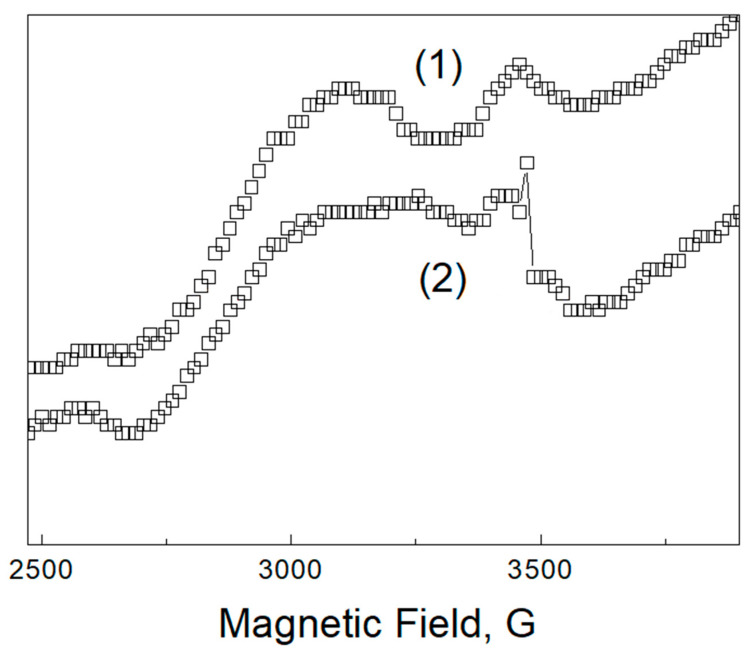
Room temperature EPR spectra of polyethylene before (1) and after temperature treatment (2). The lines in this spectrum are weaker and significantly broader in comparison with *Pd*-containing samples.

## Data Availability

Data available upon request.
